# Supplementary material for: Do children interpret informants’ confidence as person-specific or situational?
Source: PLoS One. 2024 May 8;19(5):e0298183. doi: 10.1371/journal.pone.0298183 (PMC11078414; doi:10.1371/journal.pone.0298183)
Supplement: Attachment — Submitted filename: Response to Reviewers.docx [file pone.0298183.s001.docx]

École de psychologie | School of Psychology

136 Jean-Jacques Lussier, Vanier 3002, Ottawa, ON K1N 6N5

🕿 613-562-5801

January 11, 2024

Dear Editor and Reviewers,

We would like to thank you for providing insightful comments and considering the manuscript entitled “**Do children interpret informants’ confidence as person-specific or situational?**” for publication as a **Research article** in *PLoS ONE*.

We have uploaded an unmarked version of our revised manuscript as well as a marked-up copy that highlights changes made to the original version. We also addressed the Editor’s and Reviewers’ comments. Our responses can be found below.

**Editor’s comments**

1. Please ensure that your manuscript meets PLOS ONE's style requirements, including those for file naming.

**Response**: The style requirements have been reviewed.

2. Note from Emily Chenette, Editor in Chief of PLOS ONE, and Iain Hrynaszkiewicz, Director of Open Research Solutions at PLOS: Did you know that depositing data in a repository is associated with up to a 25% citation advantage (<https://doi.org/10.1371/journal.pone.0230416>)? If you’ve not already done so, consider depositing your raw data in a repository to ensure your work is read, appreciated and cited by the largest possible audience. You’ll also earn an Accessible Data icon on your published paper if you deposit your data in any participating repository (<https://plos.org/open-science/open-data/#accessible-data>).

**Response**: We have deposited our minimal data set underlying the results of our manuscript in a public repository (available here: <https://osf.io/9a4j2/?view_only=dc7e670c756c41ceb8e84735faf397ad>). It is to be noted that upon verifying that all data supporting our findings were available, we rectified the number of participants included in Experiment 2 which slightly changed results (e.g., means, standard deviations) but does not impact our conclusions.

3. In your Data Availability statement, you have not specified where the minimal data set underlying the results described in your manuscript can be found.

**Response**: Our minimal data set will be available on the Open Science Framework upon acceptance (private link: <https://osf.io/9a4j2/?view_only=dc7e670c756c41ceb8e84735faf397ad>). We would thus like to change our Data Availability as followed: The data that support the findings of this study is available in a public repository (Open Science Framework) and can be found here [link available at acceptance].

4. We note that you have stated that you will provide repository information for your data at acceptance. Should your manuscript be accepted for publication, we will hold it until you provide the relevant accession numbers or DOIs necessary to access your data. If you wish to make changes to your Data Availability statement, please describe these changes in your cover letter and we will update your Data Availability statement to reflect the information you provide.

**Response**: We appreciate this opportunity to change our Data Availability statement and as previously indicated, we would thus like to change it as followed: The data that support the findings of this study is available in a public repository (Open Science Framework) and can be found here [link available at acceptance].

5. Please review your reference list to ensure that it is complete and correct. If you have cited papers that have been retracted, please include the rationale for doing so in the manuscript text, or remove these references and replace them with relevant current references. Any changes to the reference list should be mentioned in the rebuttal letter that accompanies your revised manuscript. If you need to cite a retracted article, indicate the article’s retracted status in the References list and also include a citation and full reference for the retraction notice.

**Response**: The reference list has been reviewed and is complete and correct. Because we had cited a paper that had been submitted but has not yet been accepted, we removed this reference (see line 610). In response to one of Reviewer #2’s suggestions, we also added 2 references (see line 639). As a result, our numbered in-text citations and references were rearranged.

**Reviewer #1**

1. My one comment is that the analyses, while they are sound as presented, could be streamlined a bit and made more parsimonious. That is, instead of the repeated variants of t-tests (or ANOVAs in the later experiment) that tackle the same underlying data in slightly different ways - the authors could opt for simply analysing the trial by trial data using a mixed effect logistic regression (this would allow for analysis of the overall tendencies and specific trial by trial data in a single model). I don't see this is a necessary change, but it could streamline the paper somewhat (if that's something the editorial team would like in this instance).

**Response**: We agree with Reviewer #1 that logistic mixed-effects models would be a desirable strategy with the present data, however we chose to keep our analyses unchanged in order to remain consistent with our pre-registrations.

2. My only suggestion for the authors is to the include confidence intervals around all estimated effect sizes - those, in line with the release of the data on the OSF, will help for future researchers wanting to build on this research (or include it in a meta-analysis).

**Response**: We have added confidence intervals around all estimated effect sizes.

**Reviewer #2**

1. Introductory section:

Line 46: I suggest better specifying the age of the samples of children investigated in the studies cited.

**Response**: We specified the age of the samples from the studies cited (e.g., see lines 49, 65 and 69).

2. Line 131: I suggest delving deeper into the explanation of Moore and colleagues' research.

**Response**: We provided additional information regarding Moore and colleagues’ research (see lines 134-143).

3. Methodology
   I suggest clarifying the methodology used (the three conditions and the different phases) through a table or a small graphic.

**Response**: We clarified our methodology by adding a table (Table 1) summarizing the

three conditions and the different phases.

4. Discussion
   To justify the results in parts different from those emerged in previous studies, I suggest adding studies that investigate the relationship between study results and memory development in childhood.

**Response**: We added information in the General Discussion on the potential relationship between our results and memory development in childhood (see page 28).

5. Finally, I suggest including a section in which to propose how these results could be useful for the purposes of structuring educational programs and technologies for the age group considered.

**Response**: We included such a section in the revised General Discussion (see page 29).

We once again thank the Editor and both Reviewers for their comments. We believe that *PLoS ONE* is the ideal outlet for our manuscript and that this research will impact further advancements in this field.

Sincerely,

**Aimie-Lee Juteau**

Étudiante au Doctorat | Doctoral Student

Psychologie Clinique | Clinical Psychology

Laboratoire sur la pensée des enfants | Childhood Thinking Laboratory

Université d'Ottawa | University of Ottawa

Elle/She/Her

136 Jean Jacques Lussier, Ottawa, ON, Canada K1N 6N5

**Email:** [ajute095@uottawa.ca](mailto:ajute095@uottawa.ca)
